# Patient-initiated switching between private and public inpatient hospitalisation in Western Australia 1980 – 2001: An analysis using linked data

**DOI:** 10.1186/1743-8462-2-12

**Published:** 2005-06-27

**Authors:** Rachael E Moorin, C D'Arcy J Holman

**Affiliations:** 1 Australian Centre for Economic Research on Health (UWA Campus), School of Population Health, The University of Western Australia, Australia; 2 Centre for Health Services Research, School of Population Health, The University of Western Australia, Australia

**Keywords:** Data linkage, Health Policy, Health Insurance, Australia.

## Abstract

**Background:**

The aim of the study was to identify any distinct behavioural patterns in switching between public and privately insured payment classifications between successive episodes of inpatient care within Western Australia between 1980 and 2001 using a novel 'couplet' method of analysing longitudinal data.

**Methods:**

The WA Data Linkage System was used to extract all hospital morbidity records from 1980 to 2001. For each individual, episodes of hospitalisation were paired into couplets, which were classified according to the sequential combination of public and privately insured episodes. Behavioural patterns were analysed using the mean intra-couplet interval and proportion of discordant couplets in each year.

**Results:**

Discordant couplets were consistently associated with the longest intra-couplet intervals (ratio to the average annual mean interval being 1.35), while the shortest intra-couplet intervals were associated with public concordant couplets (0.5). Overall, privately insured patients were more likely to switch payment classification at their next admission compared with public patients (the average rate of loss across all age groups being 0.55% and 2.16% respectively). The rate of loss from the privately insured payment classification was inversely associated with time between episodes (2.49% for intervals of 0 to 13 years and 0.83% for intervals of 14 to 21 years). In all age groups, the average rate of loss from the privately insured payment classification was greater between 1981 and 1990 compared with that between 1991 and 2001 (3.45% and 3.10% per year respectively).

**Conclusion:**

A small but statistically significant reduction in rate of switching away from PHI over the latter period of observation indicated that health care policies encouraging uptake of PHI implemented in the 1990s by the federal government had some of their intended impact on behaviour.

## Background

Coexistence of public and private health insurance, such as in Australia, has been the subject of intense debate among health economists and policy makers [1]. The main issue surrounding this debate has been how the mix of public and private health care financing influences the demand for private health insurance (PHI) and whether PHI takes pressure off the public system [2].

Falling PHI membership, observed since the introduction of Medicare in 1984, was thought to have increased the demand on the public system [3], prompting the federal government to implement policies aimed at encouraging possession of PHI to take the pressure off public hospitals and restore balance to the health care system [3-5]. Since 1995 three major policy reforms have been introduced in Australia [6]. Firstly, in 1995 selective contracting was introduced. The then federal Labor government passed legislation allowing private health plans to contract selectively with hospitals and doctors so as to improve competition on price and quality. Secondly, government subsidisation of PHI was introduced in 1997 by the Conservative coalition federal government as a means-tested rebate, capped at a flat amount irrespective of the cost of PHI, combined with an income tax surcharge for high income earners without PHI. The rebate part of the policy was subsequently replaced in 1999 by a non-means-tested 30% rebate for PHI available to everyone. Finally, in 2000, lifetime community rating was introduced. This policy relaxed the previous stringent community rating system by allowing the price of PHI to be varied according to the age at which a member joined [6].

The Australian Healthcare Agreement 1998–2003 committed the Commonwealth and states to review the relationship between PHI cover and the use of hospital services by private patients. The investigation of this relationship is becoming a priority as there is disagreement among commentators as to the financial efficiency of the 30% rebate [5,7-10]. To date, analyses of the effects of policies aimed at supporting PHI in Australia have primarily centred on changes in the proportion of the population covered by PHI [3,6,11,12]. However, changing the proportion of the population covered may not directly translate to increased utilisation and, therefore, reduce pressure on the public system. The relationship between PHI cover and type of hospital use is complex because the universality of Medicare in Australia means that anyone can be treated in a public hospital at no charge regardless of their insurance status.

The right of individuals to choose between public and private insurance, regardless of the status (public or privately insured) of previous hospitalisations or the possession of PHI is protected by the principles of Medicare as set out in the Health Insurance Act of 1973. Thus switches between use of the public and PHI system are initiated solely by patients based on choice rather than being mandated by government, notwithstanding that this 'choice' may be constrained in some instances by socioeconomic and locational access factors. We propose that since possession and utilisation of PHI are not equivalent, analysing the effectiveness of the recent government strategies in relieving the pressure on the public system cannot be accomplished by evaluations of changes in possession of PHI alone. Rather, changes in choice, as reflected by patient-initiated switching between the public and private insurance systems must be analysed.

The aim of this study was to identify and measure changes in the behavioural patterns of switching between public and privately insured status for hospitalisation by the population of Western Australia using our novel couplet methodology for analysing longitudinal data.

## Method

### Hospital morbidity data extraction and case selection

The WA Data Linkage System [13] was used to extract all hospital morbidity data system (HMDS) records from 1^st ^January 1980 to 31^st ^December 2001, containing encrypted patient identification and episode numbers, age, gender, date of admission, date of separation, payment classification (public, insured private, or "other"), and hospital type. The "other" payment category, which included the private uninsured (2.2% of the total episodes), workers compensation (1.8%), motor vehicle (0.7%), defence force personnel (0.3%) and Veteran Affairs (1.7%) classifications was removed from the data set, leaving only the categories of public and private insured. This was done because the study was concerned with elective shifts between PHI and public categories; not enforced payment classifications due to mandatory funding arrangements, or private episodes for which the patients paid the full cost.

### Data coverage

This research has made use of records from the HMDS which is the inpatient information system for WA acute care hospitals. The data collected by the HMDS are patient identification, socio-demographic, services, administration and clinical diagnosis information. Every WA hospital defined as an acute care facility has to provide data via the Health Act or for private hospitals as part of their license and every free-standing day surgery unit must also provide data. The HMDS includes patient information for all acute A class hospitals, day surgery units, geriatric and psychiatric inpatient facilities in general hospitals and, as of July 1993, healthy newborn infants born in hospital. It does not include patients of stand-alone geriatric and psychiatric institutions, geriatric hostels or rehabilitation units; however, most metropolitan hospitals have geriatric and psychiatric units attached for which data are provided for the HMDS. Over the lifetime of this study there were no significant changes in the HMDS coverage.

### Formation of episodes of care and assignment of payment classification

For each individual in the data set all eligible hospital records were grouped into episodes of care, using the separation and admission dates to define temporally contiguous periods of health care service utilisation. Thus one episode of care could have contained one or more inter hospital transfers. Each episode of care was assigned to one of the two eligible payment classifications (public or privately insured) on the basis of the initial payment classification at admission, where inter-hospital transfers were involved. Allocation of public or PHI status was based solely on payment classification and not on hospital type. This was done because the focus of this paper (consistent with the focus of public policy making in Australia) was on use of PHI rather than hospital type.

### Stratification by age group

Stratification was based on age at admission of the first episode in each couplet. Each episode of care was assigned to one of four broad age categories (0–16 years, 17–39 years, 40–69 years and 70+ years) chosen to represent PHI market segmentation (children, young adults, middle age and old age) following consultation with a local private health insurer.

### Formation and classification of hospital couplets

**Figure 1:**
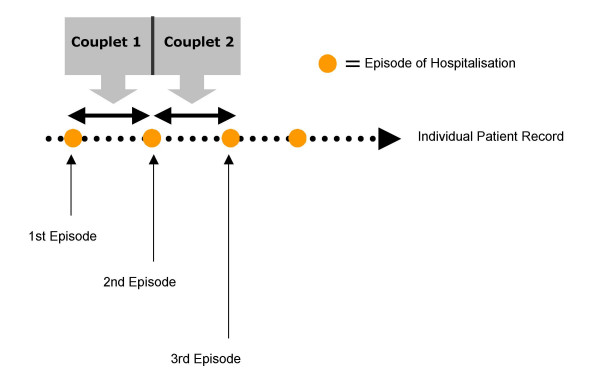
The formation of hospital couplets.

For each individual in the data set, eligible episodes of care were grouped incrementally, starting with the index (first episode of care) to form hospital couplets such that episodes 1 and 2 formed hospital couplet 1, episodes 2 and 3 formed hospital couplet 2 and so on (see figure 1). Hospital couplets were categorised depending upon the sequential combination of payment classifications of their two contributing episodes of care as follows:

(i) Concordant public couplets containing only public episodes of care.

(ii) Concordant private couplets containing only privately insured episodes of care.

(iii) Discordant (mixed) public to private couplets had the first episode of care as public and the second as privately insured.

(iv) Discordant (mixed) private to public couplets had the first episode of care as privately insured and the second as public.

### Analysis of the mean intra-couplet interval

The intra-couplet interval was defined as the time in days between the final separation from the first episode of care and admission to the second episode of care. The mean intra-couplet interval was calculated for each couplet classification by the year of admission of the second episode of care (a term we subsequently refer to as the couplet year) and expressed as a ratio of the sum of the mean intra-couplet interval of all couplets in that year, regardless of classification. We subsequently refer to this measure as the grand mean. Thus a ratio greater than one for a particular couplet classification was indicative of an intra-couplet interval longer than the grand mean for that couplet year. The couplet year indicates the year the switch (choice) was made.

### Analysis of behavioural patterns in switching of payment classification

The proportion of each type of discordant couplet relative to the total number of couplets having a first episode of care in the baseline payment classification was determined independently for all intra-couplet intervals (in whole years) in the data set.

This analysis was performed separately for each age group for the whole observation period and two predefined time periods (1981 to 1989 and 1990 to 2001) chosen to represent the two main eras of health care policy in Australia. The first related to the removal and re-introduction of free public hospital care, while the second related to changes in federal health policies aimed at supporting PHI [5,12,14,15]. Hospital couplets were partitioned into the two time periods using the year of admission of second episodes of care. The values obtained were plotted as segmented trend lines and the average rates of loss from each payment classification per year of intra-couplet interval were calculated using least squares fit.

## Results

### Characteristics of the hospital couplets in the HMDS data file

The HMDS data file contained data pertaining to 1,979,946 individuals of which 1,185,014 (60%) had at least one valid couplet. The 7,561,486 episodes of care recorded for these individuals formed 6,376,472 distinct hospital couplets (see table 1). Of these, approximately one half (51%) had an intra-couplet interval of one year or greater. Details of the distribution of the hospital couplets by intra-couplet interval are shown in table 2. Significant differences were observed in the characteristics of couplets having intra-couplet intervals of less than 1 year compared with those of one year or over. The largest differences were observed in relation to hospital type and couplet category.

**Table 1:** Characteristics of individuals, hospital episodes and hospital couplets

**Characteristic**		**Individuals**		**Episodes**		**Couplets **(2^nd ^Episode)	
		**Number**	**% of Dataset**	**Number**	**% of Dataset**	**Number**	**% of Dataset**
**Sex**	Male	522167	44.1	3156438	41.7	2634271	41.3
	Female	662841	55.9	4405035	58.3	3742194	58.7
	Indeterminate	4	0.0	13	0.0	7	0.0
TOTAL		1185014	100	7561486	100	6376472	100

**Age Group**	0–16 Years	313244	26.4	1045092	13.9	731848	11.5
	17–39 Years	443602	37.4	2495039	33.1	2051437	32.3
	40–69 Years	326974	27.6	2708572	35.9	2381598	37.5
	70+ Years	100870	8.5	1292462	17.1	1191592	18.7
TOTAL		1184690	100^1^	7541165	100^2^	6356475	100^3^

**Hospital**	Teaching	367964	31.1	2852257	37.7	2484293	39.0
**Type**	Public Metropolitan	198456	16.7	902286	11.9	703830	11.0
	Private Metropolitan	313563	26.5	1838354	24.3	1524791	23.9
	Public Country	279315	23.6	1745527	23.1	1466212	23.0
	Private Country	25097	2.1	208883	2.8	183786	2.9
TOTAL		1184395	100^4^	7547307	100^5^	6362912	100^6^

^1 ^324 missing

^2 ^20321 missing

^3 ^19997 missing

^4 ^619 missing

^5 ^14179 missing

^6 ^13560 missing

Missing records were caused by missing data in the relevant fields of the individual records and do not reflect deficiencies in the linkage process.

**Table 2:** Distribution of hospital couplets by characteristics and intra-couplet interval

**Characteristic**		**Intra-Couplet Interval (I-CI)**					
		**Less than 1 Year**			**1 Year or greater**		
		
		**Number**	**% in Dataset**	**% Across I-CI**	**Number**	**% in Dataset**	**% Across I-CI**
**Sex**							
	Male	1397957	44.6	53.1*	1236314	38.2	46.9
	Female	1739643	55.4	46.5*	2002551	61.8	53.5
	Indeterminate	5	0.0	71.4	2	0.0	28.6
TOTAL		3137605	100	49.2*	3238867	100	50.8

**Age Group**							
	0–16 Years	318255	10.2	43.5*	413593	12.8	56.5
	17–39 Years	871234	27.9	42.5*	1180203	36.5	57.5
	40–69 Years	1271495	40.7	53.4*	1110103	34.4	46.6
	70+ Years	664439	21.3	55.8*	527153	16.3	44.2
TOTAL		3125423	100^1^	49.2*	3231052	100^2^	50.8

**Hospital Type**							
	Teaching	1554708	49.6	62.6*	929585	28.7	37.4
	Public Metropolitan	249979	8.0	35.5*	453851	14.0	64.5
	Private Metropolitan	573729	18.3	37.6*	951062	29.4	62.4
	Public Country	672844	21.4	45.9*	793368	24.5	54.1
	Private Country	77205	2.5	42.0*	106581	3.3	58.0
	Other	9140	0.3	67.4*	4420	0.1	32.6
TOTAL		3137605	100	49.2*	3238867	100	50.8

**Couplet Category**							
Concordant	Public	2104333	67.1	56.7*	1605368	49.6	43.3
	Private	887622	28.3	43.0*	1176629	36.3	57.0
Discordant	Public – Private	74132	2.4	31.5*	161140	5.0	68.5
	Private – Public	71518	2.3	19.4*	296730	9.1	80.6
TOTAL		3137605	100	49.2*	3238867	100	50.8

* Statistically significantly different (at the 0.05% level) to the proportion of couplets with an intra-couplet interval 1 year or greater.

^1 ^12182 missing

^2 ^7815 missing

**Figure 2:**
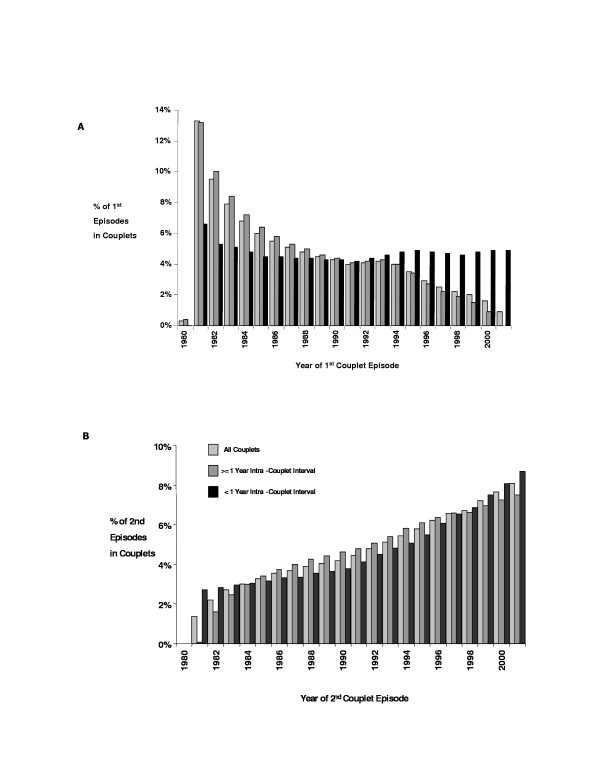
Distribution of the proportion of first and second episodes in couplets by year.

The distribution of first couplet episodes in each year of observation was more uniform in couplets with less than a one year intra-couplet interval compared with couplets longer intra-couplet interval or all couplets in the data file as shown in figure 2(A). The reduction of first couplet episodes in couplets with intervals = one year was directly proportional to the number of years remaining in which a second episode (thus completing a couplet) could be observed. The lack of first episodes observed in 1980 was the result of a reduced volume of data in the original HMDS file for that year, most likely caused by an extraction error.

Figure 2(B) shows the distribution of second episodes in couplets over the observation period. The proportion of second episodes increased over the observation period regardless of the duration of the intra-couplet interval. This was a function of the increased number of individuals eligible to complete a couplet with a second episode as time progressed.

### Mean intra-couplet interval

**Figure 3:**
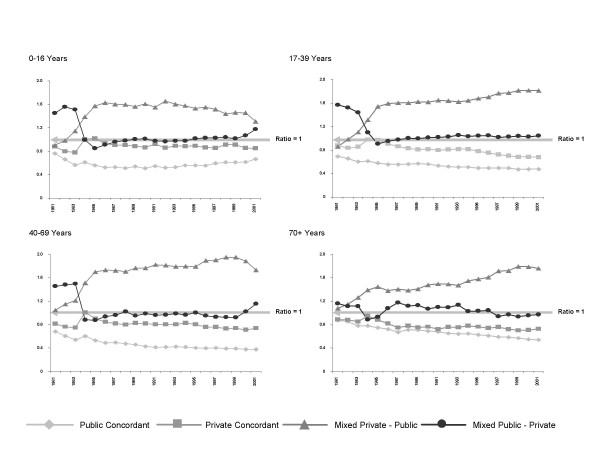
Ratio of the mean intra-couplet interval to the grand mean for each couplet category by age group and couplet year.

The ratio of the mean intra-couplet interval observed for each couplet category relative to the grand mean by age group and couplet year is shown in figure 3. Discordant couplets had the longest intra-couplet intervals, having on average a ratio relative to the annual grand mean intra couplet interval of 1.35, while concordant couplets types had the shortest intra-couplet intervals, their ratio being 0.65. The overall pattern indicated that the longer the time between the first and second episode of a couplet, the more likelihood there was of a change in payment classification, especially where the first payment classification was private. The trends also indicated that, within each age group, individuals with public concordant couplets, on average, had shorter intervals between episodes (ratio 0.5) than individuals with private concordant couplets (ratio 0.8).

The removal / re-introduction of free public hospital care (1980 – 1984) has previously been shown to have had a significant effect on use of in-patient insurance classifications [16]. In this study no significant difference was observed with regard to when the first episode of a couplet occurred (pre or post Medicare); however, the point in time of the second episode did correspond with behavioural change as can be seen in figure 3.

Differences in trend were observed across the four age groups. The 0–16 years age group trended towards a reduction in the intra-couplet interval associated with discordant private to public couplets after 1985. This was not observed during the late 1980s and early 1990s in the other three age groups. The 40–69 years age group showed a trend pattern similar to the 17–39 years age group until 2000, when the average intra-couplet interval associated with discordant private to public couplets reduced sharply. In addition there was a sharp increase in the average intra-couplet interval associated with discordant public to private couplets (also observed in the 0 to 16 years age group) observed at that time. In the oldest age group (70+ years) the average difference in intra-couplet interval between public and private concordant couplets was much smaller than observed in any of the other three age groups.

Over all age groups the trend in concordant couplets was that of a slowly reducing average intra-couplet interval, excluding the 1983 – 1984 period, where the average intra-couplet interval for private concordant couplets increased in all age groups.

### Behavioural patterns in switching of payment classification

Differences were observed in the rate of loss from the public and privately insured payment classifications across age groups as shown in table 3. Across all age groups the largest losses, at all intra-couplet intervals, occurred from the privately insured payment classification. The largest differences in the rates of loss were observed in the 70+ years and the 17–39 years age groups, where an additional 2.39 and 1.78 percent of privately insured episodes, respectively, were lost for every year of intra-couplet interval. The rates of loss from the privately insured payment classification over shorter intra-couplet intervals (defined as 0 to 13 years) were greater than the rates of loss over longer intra-couplet intervals (defined as greater than 14 years) in all age groups with the largest difference being observed in the 40–69 years age group and the smallest in the 70+ years age group. The definition of short and long intra-couplet interval was based on an observed substantial change of slope (inflection) in the segmented trend lines.

**Table 3:** Loss from each payment classification as a function of age and intra-couplet interval

	**Rate of Loss (percent per intra-couplet year)**				**Difference in Rate (percent per intra-couplet year)**	
	
	**Public**	**Private Insured**			**Public vs. Private**	**Private Insured**
	
Age group*	All intervals*	All intervals*	**0 to 13 yrs**	**14 to 21 yrs**	All Intervals*	**0 to 13 vs. 14 to 21 yrs**
**0–16 Years**^$^	0.63	1.77	2.37	0.40	1.14	1.97
**17–39 Years**	0.49	2.27	2.39	0.55	1.78	1.84
**40–69 Years**	0.81	1.94	2.56	0.46	1.13	2.10
**70+ Years**	0.25	2.64	2.67	1.91	2.39	0.76

* Averaged over all intra-couplet intervals in years.

^$^Maximum intra-couplet interval 16 years as age determined at second episode.

**Figure 4:**
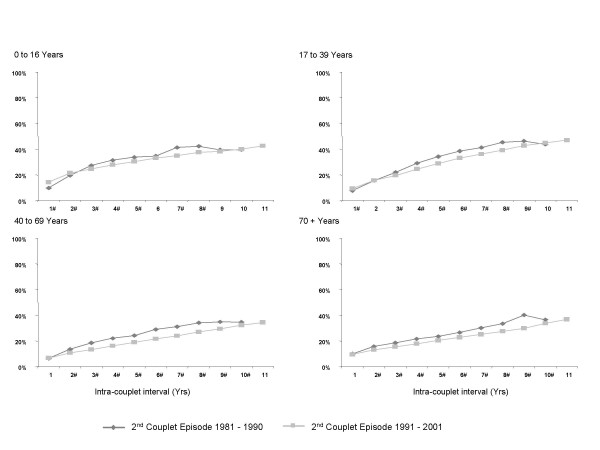
The proportionate discordance among hospital couplets with a private first episode by decade of the second couplet episode. # Significant difference (p < 0.01) between the percentage of discordant second episodes occurring in 1981–1990 versus 1991–2000.

Figure 4 shows the degree of switching from private to public episodes over the two designated eras in health care policy. In all age groups the average rate of switching away from the private sector in 1991–2001 (era 2) was lower than that observed in 1981–1990 (era 1). The decrease in rate was small (average across all age groups -0.35% per intra-couplet year) but statistically significant. In addition, significance testing of the difference between each pair of proportions (era 1 versus era 2) indicated a significant difference for the majority as indicated in figure 4.

We also found that as the intra-couplet interval increased, the difference between the proportions of discordant couplets having a private first episode versus a public first episode increased from 4.2% at one year to 34.3% at 18 years (data not shown). This indicated that overall, regardless of age, there was greater switching away from PHI than away from the public classification.

## Discussion

As expected, due to their greater capacity to move between respective payment classifications, private patients were found to be more likely to switch payment classifications in their next admission than public patients, irrespective of the length of time between the two episodes. There are a number of possible explanations for this including putative structural and cognitive reasons for the observed behaviour. Structurally, the average patient who begins with a public classification is likely to be of lesser socioeconomic means than the average patient who begins with a private insured classification. The option of the former patient to use PHI at the next hospitalisation will be dependant upon them taking out, or at the very least maintaining (if they had private cover at the initial episode but did not use it) private cover in the meantime. The same pre-requisite does not exist for an initially privately insured patient accessing a public classification on the next occasion. Also patients with private insurance experiencing trauma or an acute disease event may, in some circumstances, be admitted in an emergency as a public patient, thus to some extent disallowing the patient from exerting their preference. Cognitive explanations include the possibility that PHI may not be as entrenched culturally as Medicare (the public system) in this population and, as a consequence, use of PHI may be more dependant upon marketed value propositions than use of Medicare. In other words, PHI may be perceived by the populace as a market good, whereas the public system (Medicare) may be perceived as a fundamental right.

We also found that the degree of switching from PHI towards the public system was inversly proportional to the length of time between episodes. Possibly, healthier individuals were more likely to switch to the public system than sicker individuals, assuming that a relatively short duration of intra-couplet interval can be taken as an indicator of increased morbidity. This phenomenon is consistent with reports that the decline in the proportion of the eligible population holding PHI since the introduction of Medicare in 1984 has been largely attributed to younger and healthier individuals dropping out [11,12]. This finding indicated the presence of a substantial cross-subsidisation between low risk and high risk individuals further exacerbating the adverse selection price 'death spiral' of PHI [11]. Improving the risk profile of PHI holders has been a major focus of recent federal government policy, with the introduction of a lifetime community rating in 2000 in an effort to encourage younger, healthier individuals to take out and remain in PHI funds. Our finding that in all age groups the overall rate of switching away from PHI was slightly higher in the period 1981 – 1990 compared with the rate in the period 1991 – 2001, suggests that these policies have had an effect on behaviour consistent with the government's intention.

An alternative, but less likely explanation for our findings, may be systematic differences in the cognitive decision making process between long intra-couplet intervals compared with between shorter intra-couplet intervals. For example, individual historical preferences may play a role in short interval switching but may not be important over longer intervals, where decisions may be made in isolation. While it is important to consider this alternative, we feel that if the changes observed in switches away from PHI were largely due to differences in cognitive decision making, one would expect to see a similar phenomenon in switching away from a public classification. Such a phenomena was not observed in our data.

In all age groups, our analysis indicated that the overall rate of switching from the private payment classification was slightly higher in the period 1981 – 1990 compared with the rate in the period 1991 – 2001, suggesting that the policies had an effect on behaviour consistent with the government's intention.

### Assumptions and limitations of the approach

This study made use of the WA Data Linkage Project which is unique in Australia and is one of only a small number of population-based record linkage systems in the world. The use of administrative data has strengths and weaknesses. For example data can be inaccurate due to recording or coding errors [17] or linkage errors. For this study individual patient records were linked by probabilistic matching, using an automated computer algorithm based on the probability of two records being from different people having the same identifier and two records from the same person having different identifiers. The probabilities were then aggregated into a score and checked against a threshold to determine if a match was made. This technique typically has been found to have a true positive predictive value of 95–99% and a negative predictive value of 98–99% [18]. Extensive validation of the quality of the performance of matching has been undertaken on the WA Record Linkage Project using sampling techniques and the proportions of mismatches and missed matches found were in the order of approximately 0.11% [18].

Linked data have the advantage of supporting a large and diverse research programme at relatively low cost, once the infrastructure is in place. They have the capacity to provide a population-based view of events experienced longitudinally by individuals across all institutions [17]. Given the objectives of this study, the latter point makes the use of linked data particularly appropriate.

The approach we have taken in order to analyse the use of PHI and Medicare by the population of Western Australia is unique in at least two respects. Firstly the study was conducted at the population level, due to the use of hospital morbidity data. Therefore, the "reference population" was not merely an abstract concept as in conventional quantitative research, but an operationalised descriptor of the study sample. Secondly, our 'couplet methodology' has not been reported previously in the literature and represents a new method of analysing longitudinal data on use of health insurance. The couplet methodology has enabled patterns to be measured based the behaviour of individuals rather than average shifts in private – public mix generated from unlinked episodes of care. However, we recognise that since the analysis was based on episodes of care, patient initiated switching as part of a hospital transfer could not be analysed. This limitation may have affected our conclusions about intra-couplet intervals.

This paper is dedicated to explaining the couplet technique for the first time and applying it to address an initial set of relatively descriptive questions (ie teasing out what is happening). The next stage of investigation is a more analytic analysis which recognises that a wide range of potential explanatory variables might predict the couplet-based phenomenon of switching (the why). For example hospitalisation rates in set periods before and after a couplet, stratified by different lengths of stay and/or different DRG weights. In addition, the admission types (emergency/elective) of the members of the couplet may also be important predictors. We feel that the couplet methodology described in this paper will enable significant inroads to be made into the investigation of such explanatory variables.

## Conclusion

Our study found that the population of Western Australia exhibited distinct behavioural patterns in the switching of payment classifications for inpatient hospitalisation between 1980 and 2001. Private patients were more likely to switch payment classification than public patients with shorter intervals between episodes corresponding to a greater probability of private-to-public switching. However, the average rate of switching from a privately insured classification was greater between 1981 and 1990 than between 1991 and 2001, indicating that recent health care policy reforms implemented by the federal government to promote uptake of PHI have had an impact on behaviour.

## Competing interests

Professor D'Arcy Holman is an independent director of HBF Health Funds inc which is the largest provider of private health insurance in Western Australia.

## Authors' contributions

The manuscript has been read and approved by all authors and the requirements for authorship have been met as outlined below. REM was responsible for the conception and design of the study; analysis and interpretation of the data; and drafting and revising the paper. CDJH was responsible for conception and design of the study; interpretation of the data; and revising the paper.

## References

[B1] Costa J, Garcia J (2003). Demand for Private Health Insurance: How Important is the Quality Gap?. Health Economics.

[B2] Cromwell D (2002). The Lore about Private Health Insurance and Pressure on Public Hospitals. Australian Health Review.

[B3] Deeble J (2003). The Private Health Insurance Rebate: Report to State and Territory Health Ministers.

[B4] McAuley IA (2004). Stress on Public Hospitals – Why Private Insurance Has Made it Worse.

[B5] Duckett SJ, Jackson TJ (2000). The New health Insurance Rebate: An Inefficient Way of Assisting Public Hospitals. Medical Journal of Australia.

[B6] Willcox S (2001). Promoting Private Health Insurance in Australia. Health Affairs.

[B7] Access Economics (2002). Striking a Balance: Choice, Access and Affordability in Australian Health Care. APHA.

[B8] Harper I (2003). Preserving Choice: A Defence of Public Support for Private Health Care Funding in Australia. Medibank Private.

[B9] Hagan P, Econotech Pty Ltd, Harper Associates (2004). Easing the Pressure: The Intergenerational Report and Private Health Insurance. Medibank Private.

[B10] Gross P (2004). The Value Proposition for Private Health Insurance and the Private Health Sector in Australia: A Framework for Public Debate about Choices.

[B11] Butler J (2002). Policy Change and Private Health Insurance: Did the Cheapest Policy do the Trick?. Australian Health Review.

[B12] Cormack M (2002). Private Health Insurance: The Problem Child Faces Adulthood. Australian Health Review.

[B13] Holman CDJ, Bass AJ, Rouse IL, Hobbs MST (1999). Western Australia: Development of a Health Services Research Linked Database. Aust NZ J Public Health.

[B14] Blewett N (2000). The Politics of Health. Australian Health Review.

[B15] Duckett SJ (2004). The Australian Health Care System.

[B16] Moorin R, Holman CDJ (2004). A longitudinal study of in-patient insurance classification in Western Australia using linked hospital morbidity data.

[B17] Armstrong BK, Kricker A (1999). Editorial: Record Linkage – A Vision Renewed. Australian and New Zealand Journal of Public Health.

[B18] Holman CDJ (2002). The Analysis of Linked Health Data: Principles and Hands-On Applications.

